# Which Aortic Valve Can Be Surgically Reconstructed?

**DOI:** 10.1007/s11886-021-01525-z

**Published:** 2021-07-02

**Authors:** Karen B. Abeln, Christian Giebels, Tristan Ehrlich, Jan M. Federspiel, Hans-Joachim Schäfers

**Affiliations:** Department of Thoracic and Cardiovascular Surgery, Saarland University Medical Center, Homburg, Saar Germany

**Keywords:** Aortic valve reconstruction

## Abstract

**Purpose of Review:**

Preservation or repair of the aortic valve has evolved dynamically in the past 20 years. It leads to a high freedom from valve-related complications if an adequate valve durability can be achieved; it may possibly also improve survival. To date, little structured information is available about which valves can be repaired and which should better be replaced.

**Recent Findings:**

For surgical decision-making, the size of the aortic root is important and the anatomy of the aortic valve must be considered. In the presence of root aneurysm, most tricuspid and bicuspid aortic valves can be preserved. In aortic regurgitation and normal aortic dimensions, the majority of tricuspid and bicuspid aortic valves can be repaired with good long-term durability. In bicuspid aortic valves, the morphologic characteristics must be taken into consideration. Unicuspid and quadricuspid aortic valves can be repaired in selected cases. Generally, cusp calcification is a sign of a poor substrate for repair; the same is true for cusp retraction and cusp destruction due to active endocarditis. They are associated with limited valve durability.

**Summary:**

Using current concepts, many non-calcified aortic valves can be repaired. Modern imaging, in particular three-dimensional transesophageal echocardiography (TEE), should be able to define repairable aortic valves with a high probability.

## Introduction

### Why repair? Aortic regurgitation

Aortic valve disease is the most frequent type of all heart valve pathologies [1]. The most frequent is aortic stenosis; 13.3 % of all left-sided heart valve disease is aortic regurgitation. Irrespective of the presence of aortic valve dysfunction, the aortic valve may have to be addressed in order to treat aortic aneurysm involving the root. Traditional treatment of aortic valve disease has been surgical valve replacement. In the treatment of root aneurysm, combined replacement of valve and root has been the standard approach for 50 years [2].

Aortic valve replacement, either isolated or in combination with root replacement, has long been a reproducible procedure with low perioperative morbidity and mortality [3, 4]. With mechanical valve substitutes, however, it exposes patients to a lifelong risk of valve-related morbidity, including thromboembolic complications, anticoagulation-related hemorrhage, and prosthetic valve endocarditis [5]. In addition, there is also a low, but relevant risk of valve-related mortality [6•]. Biological prostheses do not require lifelong anticoagulation; however, they expose patients to valve-related morbidity and mortality [7]. Thus, both replacement options are associated with excess mortality, which is clearly suboptimal.

Aortic valve repair was initially designed to treat patients with aortic root aneurysm and presumably normal cusps [8, 9]. At a similar time, first attempts were made at treating isolated aortic regurgitation by repair [10, 11]. With increasing knowledge of the normal anatomy of the aortic valve and repair options, both forms of surgery have progressed in the past 20 years [12•]. Because both cusps and root each represent parts of a functional unit, the surgical strategies for valve repair and valve-preserving surgery have merged into a common concept.

By now, aortic valve repair has become a routine procedure in experienced centers, and a relevant proportion of aortic valves can be treated by reconstructive approaches. Rather than improvising an operation, repair has changed into a systematic approach with clear identification and subsequent correction of the factors involved in regurgitation.

Repair has been shown to result in a very low incidence of valve-related complications (freedom from all valve-related complications at 10 years: 88%; linearized: 1.8% per patient year), with repair failure being the most frequent [13]. Preliminary results also indicate that repair may result in better survival than replacement [14].

It has therefore become important to identify aortic valves that are repairable, and at the same time to recognize those pathologies that do not allow for a durable result (Table 1). This review intends to summarize the current knowledge on repairable aortic valves and to emphasize the pathologies that should probably better be treated by replacement.

**Table 1:** Typical pathologies amenable to repair and those better treated by replacement

Pathologies amenable to repair	
Tricuspid/bicuspid aortic valve + root dilatation + root dilatation and cusp prolapse + cusp prolapse + cusp perforation	
Tricuspid aortic valve with fenestration	
(Unicuspid aortic valve with absent calcification)	
Quadricuspid aortic valve	
Pathologies better treated by replacement	
Any valve + retraction + calcification + multiple/large fenestrations	
Active endocarditis	

### Why repair? Root aneurysm

Every root aneurysm requiring surgery also needs concomitant surgery of the aortic valve. Aortic dimensions requiring surgery are adequately summarized in current guidelines [15]. The cut-off for root diameters requiring replacement are not as well defined. The presence of relevant aortic regurgitation most likely justifies a lower size threshold for root replacement if repair is performed.

Valve-preserving root replacement was initially designed for normal tricuspid aortic valve anatomy and lesser degrees of regurgitation [8, 9]. This assumes that regurgitation was caused by aortic dilatation, and normalization of aortic dimensions would lead to normalized aortic valve configuration and function. Concomitant procedures on the aortic cusps were not performed. It was later recognized that cusp prolapse could coexist with root dilatation in about half of the cases of root aneurysm [16]. The additional correction of cusp prolapse did not increase the risk profile of the procedure; it resulted in improved mid-term function of the valve [17].

**Fig. 1:**
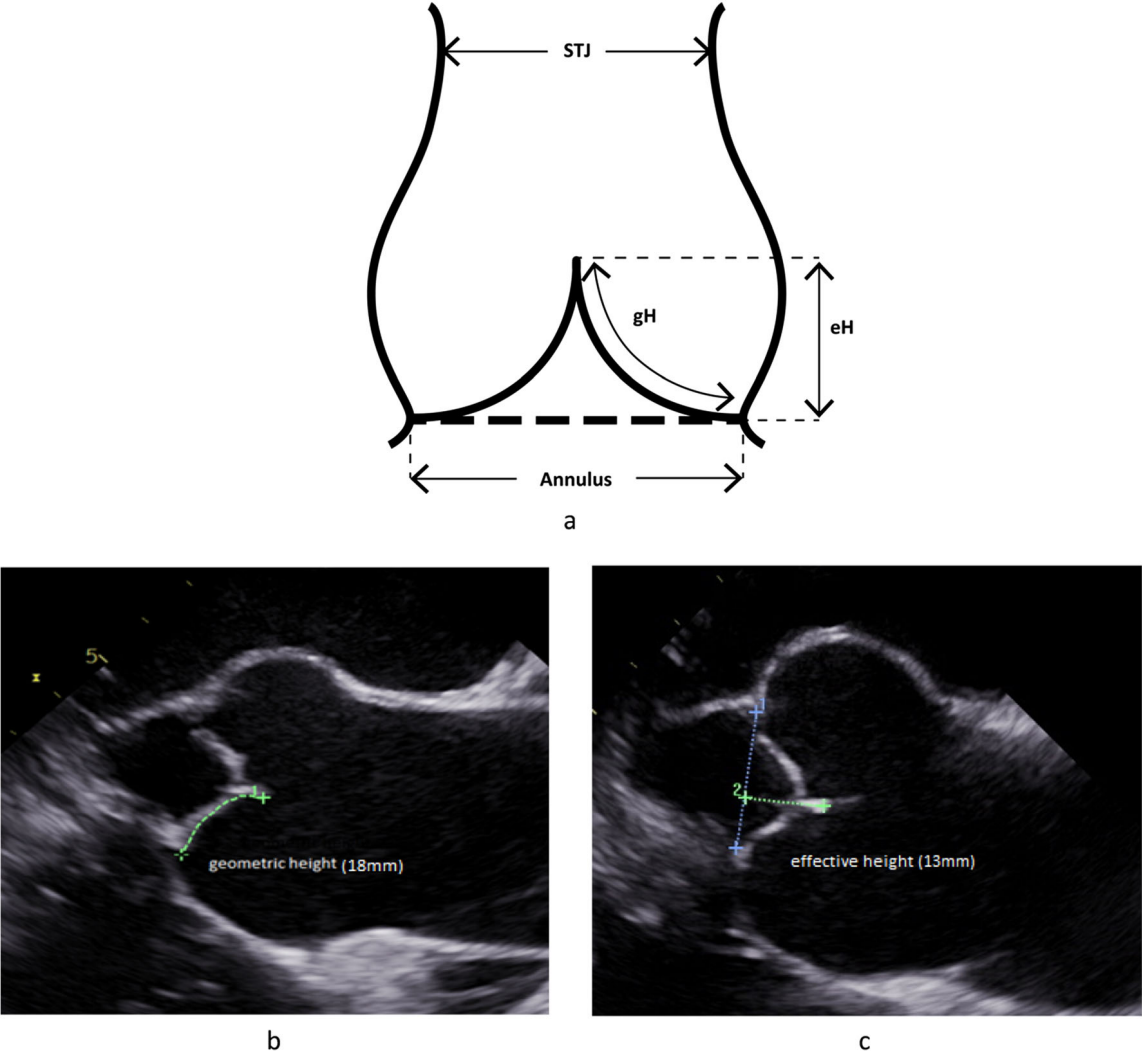
**a** Geometric concept of important cusp dimensions in assessing valve form. The cusp needs sufficient tissue height (gH: geometric height), and the free margin has a characteristic height above the annular plane (eH: effective height). **b** Long axis view of TEE prior to aortic valve repair. The geometric height of the right cusp is determined by measuring the “length” from aortic insertion to free margin; in this case, it is 18 mmm. **c** Measurement of the effective height (eH). The annular plane is marked and the distance between free cusp margin and annular plane is measured; it is 13 mm

With increasing knowledge of normal aortic valve configuration, in the introduction of the effective height concept (Fig. 1a; [18]), it was realized that prolapse is indeed present in a large proportion of patients with root aneurysm. Prolapse may be present preoperatively (commonly indicated by eccentric regurgitant jet morphology) or unmasked by the normalization of root dimensions, that is, reduction of intercommissural distance [12•]. This prolapse can be corrected reproducibly (usually by plication of the redundancy of cusp tissue) and with good long-term results [19•]. In effect, valve-preserving root replacement has been shown to result in an excellent 15-year durability (94% freedom from reoperation; 86.5% freedom from aortic regurgitation >2) if done with a tricuspid aortic valve and concomitant systematic detection and correction of cusp prolapse [19•].

Subsequently, it was shown that the concept of valve-preserving root replacement could also be applied to bicuspid aortic valves [20]. Even more frequent than with tricuspid aortic valves, cusp prolapse correction is necessary in bicuspid aortic valves, and this can be done with good long-term valve durability [21]. Calcific stenosis of the valve occurs infrequently beyond the first 10 postoperative years [22••]; it is more frequent if limited cusp calcification was present at the time of surgery. Interestingly, the addition of a pericardial patch as part of the cusp repair is associated with an increased risk of valve failure [22••].

More recently, the concept of root replacement has also been applied to the rarer variant, the unicuspid aortic valve morphology [23]. At this time, data on mid- and long-term durability of this approach are still pending.

### When not to repair? General considerations

As indicated by common sense, such a procedure has not been applied to and is not useful in calcific aortic stenosis with concomitant root dilatation but only in the presence of pliable and adequately opening cusps. Concomitant cusp prolapse is no obstacle to repair with good durability if objective intraoperative assessment of cusp configuration is performed [18, 24].

There is limited published information on the proportion of valves that can be preserved using this form of surgery. The only publication that allows for indirect estimation of the percentage of preserved or reconstructed aortic valves comes from a pioneering center [25]. An interpretation of the published data [26] indicates that only approximately a third of the patients with regurgitation and root aneurysm were treated by valve preservation [25]. Unfortunately, the authors were vague about reasons for replacement. In our own experience (unpublished data), we have been able to preserve more than 80% of aortic valves in root aneurysm, irrespective of the degree of preoperative regurgitation. The most frequent reasons for replacement were cusp retraction, variable degrees of cusp calcification, and complex cusp fenestrations.

### When to repair? General considerations

The decision for surgery in isolated aortic regurgitation is defined by current guidelines [27]. More recent data indicates that the threshold of systolic function parameters (for example, left ventricular end-systolic diameter) may be too high and imply decreased postoperative survival. An LVESD of 21 to 22 mm/m^2^ has been propagated as the more appropriate trigger for surgical treatment [28•].

As a rule, aortic valve repair is an option for aortic regurgitation in the presence of pliable cusp tissue. Regurgitation may occur in conjunction with all forms of aortic valves, with tricuspid and bicuspid being the most frequent. In addition, regurgitant unicuspid and quadricuspid aortic valves may be repaired, even though these valves have to be looked at separately. Unicuspid aortic valves may also present with predominant stenosis; repair may also be an option for this variant. The goal of repair is a durable result that depends on anatomic details. Thus, the morphology of the aortic valve is an important aspect in the decision-making process.

## Specific Considerations

### Tricuspid Aortic Valves

The mechanisms of aortic regurgitation in tricuspid aortic valves may involve moderate root dilatation and cusp pathology. Of the root dimensions, annular (>27 mm) and sinotubular (> 30 to 35 mm) dilatation appear to be the more important. Cusp pathologies include prolapse, retraction, perforations after healed endocarditis, or, rarely, commissural disruption. Fenestrations usually do not lead to aortic regurgitation; the lack of tissue in the pericommissural area, however, increases local stress and may result in elongation or rupture of the thin strand of tissue representing the extension of the free cusp margin, resulting in prolapse. Finally, active endocarditis results in cusp destruction. At this time, precise data on the relative frequency of the different pathologies are lacking.

Annular dilatation can be corrected reproducibly by annuloplasty [29] and sinotubular dilatation by tubular ascending aortic replacement or annuloplasty at the sinotubular level [30]. Cusp prolapse is easily corrected by plication of the redundant tissue [31]. If prolapse is caused by fenestration, its correction best involves closure of the fenestration with a tissue patch in order to correct the prolapse and normalize stress distribution [32]. Similarly, cusp perforations can easily be closed with a patch, at least as long as they do not involve the free cusp margin. The correction of retraction requires cusp augmentation with a patch of pericardium, and commissural disruption or retraction have to be treated by a commissural reconstruction, which is relatively complex.

Compared to valve-sparing root replacement, fewer data are available on the mid- and long-term results of isolated aortic regurgitation in tricuspid aortic valve morphology. The correction of cusp prolapse leads to good mid-term durability, also in our own experience [30, 31]. The concomitant treatment of a fenestration does not impair mid-term stability [32, 33•]. In the presence of cusp retraction, however, an increased incidence of valve failures requiring reoperation has been observed [33•, 34]. Similarly, commissural reconstruction is associated with decreased valve durability [33•].

#### When not to repair?

So far, repair has not been applied to calcific tricuspid aortic valves but only in those with pliable cusps. The results of repair in active endocarditis have shown an increased risk of reoperation, indicating that this pathology should better be replaced [35]. Owing to limited repair durability, the presence of cusp retraction indicates that the valve should be replaced rather than repaired.

To our knowledge, no data have been published regarding the frequency of repair in tricuspid aortic valves. In our experience, repair has been possible in more than 50% of regurgitant tricuspid aortic valves. The most frequent reasons for replacement have been cusp retraction, cusp calcifications, and active endocarditis. In diagnosing retraction more objectively, the determination of geometric cusp height [36] has been helpful; this can be done intraoperatively and preoperatively (Fig. 1a, b). This is best done using three-dimensional transesophageal echocardiography (TEE) [37••]. The presence of multiple large or complex fenestrations may impair repair durability and should better be treated by replacement.

### Bicuspid Aortic Valves

The mechanism of regurgitation usually involves prolapse of the fused cusp. Annular dilatation is also almost invariably present. Typically, the regurgitant jet is eccentric and directed away from the fused cusp, that is, in right/left fusion, and toward the anterior mitral leaflet. On occasion, the raphe may exhibit calcification. In a certain proportion of cases, the non-fused cusp may also be prolapsing. A variable degree of root dilatation may be present. It is assumed but uncertain whether sinotubular dilatation contributes to loss of coaptation and regurgitation.

Correction of regurgitation consequently consists of correction of cusp prolapse. In addition, annuloplasty is usually necessary. Limited calcification of the raphe can be excised with subsequent readaptation of cusp tissue.

For isolated repair of the bicuspid aortic valve, not only mid- but also long-term results are available [22••, 38••]. An annuloplasty has been shown to be an important prerequisite for a stable repair; similar findings have been made for the complete correction of prolapse, best guided by measurement of effective height [10]. Importantly, commissural orientation has been shown to be an important predictor of repair durability [39], with the best stability obtained for symmetric bicuspid aortic valves [39, 40]. Asymmetric and very asymmetric valves—as judged according to commissural orientation [22••, 38••, 41••, 42•]—have poorer durability, and increased systolic gradients may be the result of repair. Most importantly, the need for insertion of a pericardial patch into any of the cusps, whether it is for closure of fenestrations, perforations, or defects after excision of calcified cusp tissue, is associated with poor long-term durability [22••, 38••].

#### When not to repair?

As for any aortic valve, repair is not an option in the presence of marked calcification. Using the systematic application of standardized repair principles, approximately 90% of non-calcified bicuspid aortic valves have been repairable [43••]. In our own experience, reasons for replacement have been calcification extending beyond the raphe, active endocarditis, and commissural pathology [43••, 44••]. The presence of asymmetric commissural orientation [41••, 42•] will add to repair complexity and may thus contribute to a decision for replacement rather than repair.

### Unicuspid Aortic Valves

The mechanism of valve dysfunction is more complex in unicuspid aortic valves; it commonly involves a component of right cusp hypoplasia and the hypoplasia of its two commissures with the fusion of cusp tissue adjacent to the involved commissures. It may present as predominant stenosis, combined dysfunction, or regurgitation. Because of the pathological anatomy, the regurgitant jet is usually central. With increasing patient age, the right cusp will calcify, and this calcification progresses over time until most of the valve will be involved.

Since the pathological anatomy is the key problem, repair of the valve requires the conversion of valve design as part of the repair correction. Such a repair consists of converting the valve into a bicuspid or a tricuspid aortic valve by creating new commissures and adding a pericardial patch to the native cusp tissue to accommodate its regional lack. Of the two approaches, the bicuspidization has been shown to be more reproducible. This repair corrects regurgitation and improves valve opening.

So far, only mid-term results of such a repair are available [45•]. The early function of the valve has generally been good but degeneration of the pericardium used as cusp replacement has been the limiting factor for valve stability with a mean durability of slightly more than 10 years [46]. With other material, such as expanded polytetrafluoroethylene, the results have been worse [47]. The need for large patches to replace more extensive cusp calcification has been associated with a higher failure rate. Thus, repair is mainly an option in children, adolescents, and young adults in whom calcification is either minimal or absent and in whom a valve replacement is less optimal.

#### When not to repair?

In our experience, repair has been possible in more than 90% of instances in these young patients. Unicuspid aortic valves should not be repaired if the patients are older than 30 to 35 years of age, and if relevant calcification is present.

### Quadricuspid Aortic Valves

Quadricuspid aortic valves are frequent in truncus arteriosus but are very rare in a normally developed aorta. They commonly develop regurgitation in the 4th to 6th decade of life [48]. The mechanism of regurgitation appears to relate to the additional commissure, possibly also to dilatation of the sinotubular junction with a central jet and lack of central coaptation [49]. The central cusp margins are commonly thickened and fibrotic.

Different repair approaches have been proposed but not all have been successful [50–53]. The most reproducible corrections have consisted of the reduction of the sinotubular diameter and the conversion of the valve into a tricuspid or bicuspid design through detachment of one or two commissures [54, 55].

Little is known on mid- or long-term results. The lack of addressing sinotubular dilatation and design conversion have been associated with an increased probability of failure [50–53]. Likewise, little is known on specific pathologies (beyond calcification and retraction) that require replacement per se.

In our experience, most quadricuspid aortic valves have been amenable to repair and we have chosen replacement only in the rare incidence of repair failure.

## The Role of Imaging in Preoperative Decision-making

**Fig. 2:**
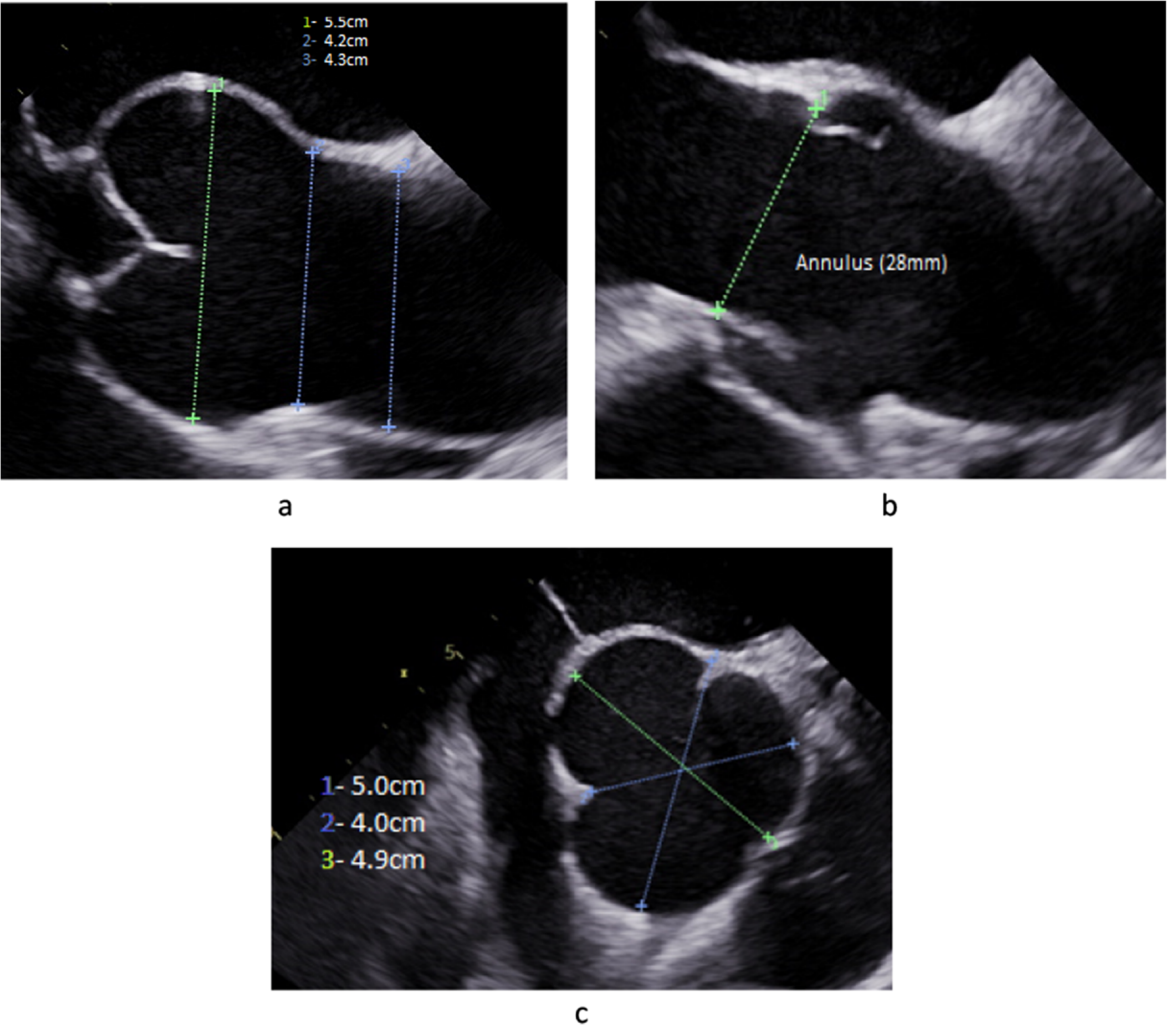
Long axis view of TEE prior to aortic valve repair. **a** The important aortic dimensions are measured in diastole. **b** Annular diameter is determined in systole. **c** Sinus dimensions are important for decision making for or against root replacement. The diameter determined in a long axis view should be controlled by measurement in a short axis view

Since the most important pathologies that prevent successful aortic valve repair are cusp-related, preoperative imaging must focus on the cusps of the aortic valve. In order to define the extent of *root dilatation*, precise measurement of the relevant aortic dimensions is necessary (Fig. 2a). In measuring the annular diameter, it is important to keep in mind that the shape may be oval, and its dimensions are largest in systole. It is therefore important to measure the annulus in systole (Fig. 2b); determination of the area will compensate for the possibility of oval shape, even though current norms are generally one-dimensional. Sinus and sinotubular diameters are determined in diastole, ensuring that the echocardiographic plane is in the center of the aorta. Ideally, measurements made in a long axis view should be double-checked by short axis projections (Fig. 2c).

**Fig. 3:**
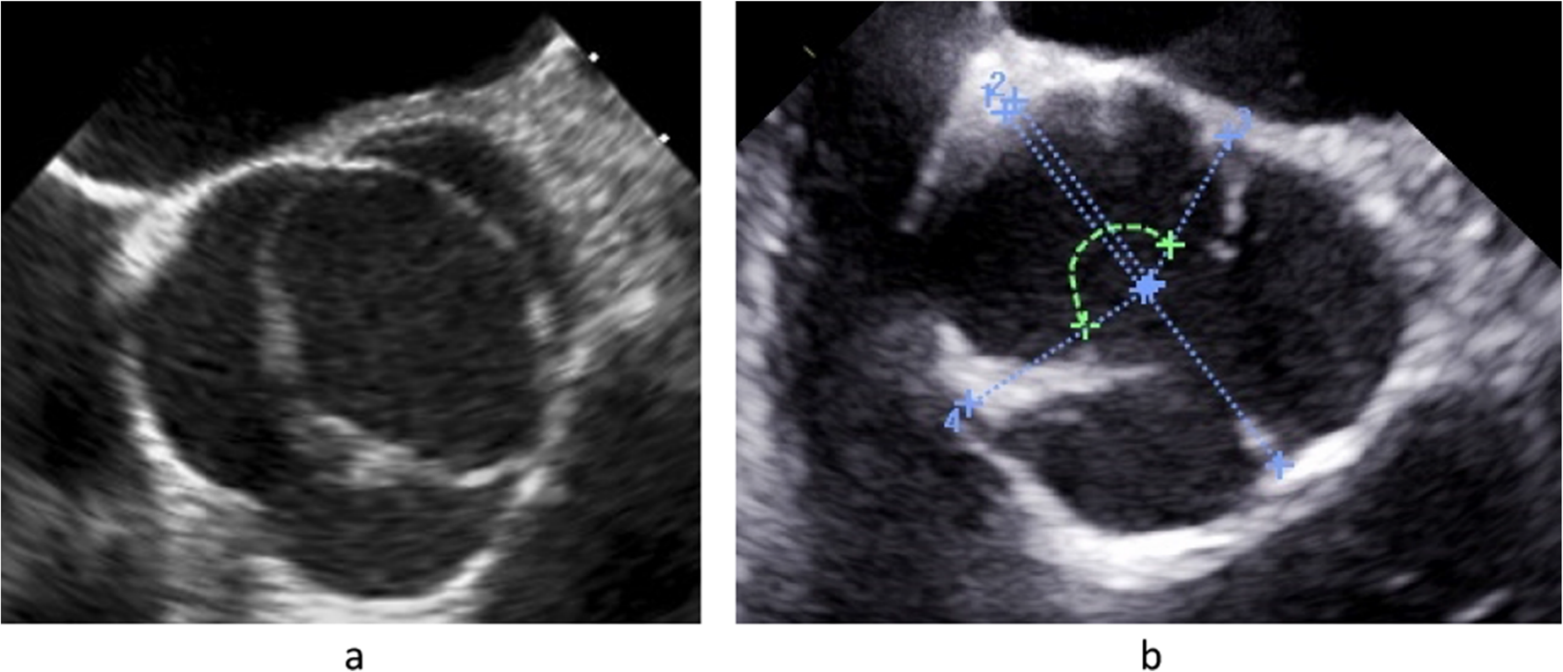
**a** TEE of an asymmetric bicuspid aortic valve with fusion of right and noncoronary cusps. The circumference of the fused sinuses encompasses more than 60% of total circumference with an angle of the nonfused cusp of 145°, classifying the valve as asymmetric bicuspid aortic valve (41). **b** In order to classify the bicuspid aortic valve correctly (41, 42), commissural orientation is determined. In a short axis view of the TEE, the center of the root is marked (42). Lines are drawn from the center to the two functional commissures, and the angle is measured for the nonfused cusp. In this case, the angle is 170°, classifying it as a symmetric valve

It is important to define the aortic valve morphology clearly because each anatomic subtype will require a specific repair approach. This should be done in a short axis view by analyzing both systolic and diastolic valve morphology. The definition may be easy in many instances of tricuspid or bicuspid anatomy. There are, however, transitions between tricuspid and bicuspid morphology, that is, bicuspid aortic valves that have a commissural orientation close to 120° [41••]. These valves require a specific and tailored approach and have to be distinguished from tricuspid aortic valves. This distinction is ideally made if the valve with its commissural orientation is not only analyzed in diastole but also in systole. The incomplete opening in the area of the rudimentary commissure will indicate the fusion associated with bicuspidity. The variable commissural orientation seen in a bicuspid aortic valve [41••] must be defined for planning of the individual repair strategy (Fig. 3a, b).

**Fig. 4:**
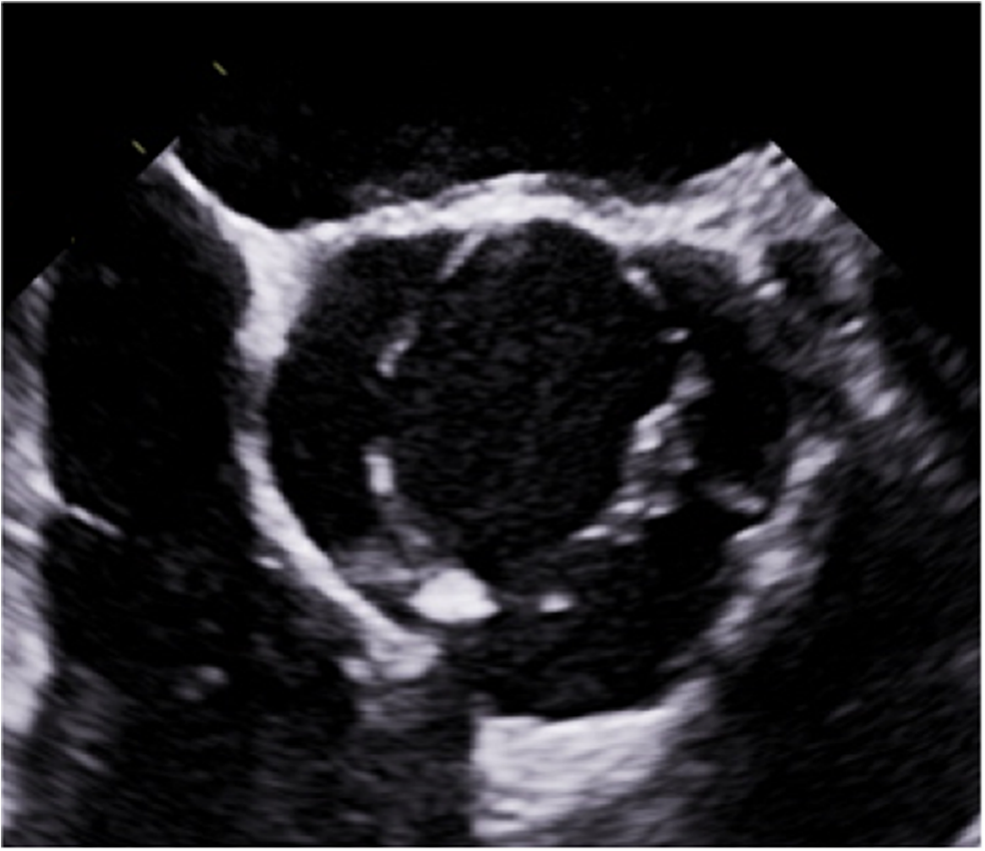
TEE short axis view of a unicuspid aortic valve. Typically, the valve opens fully in only one commissure (in this case the left/noncoronary commissure). The two other commissures are rarely visible, and valve opening does not reach the commissures fully, indicating cusp fusion

**Fig. 5:**
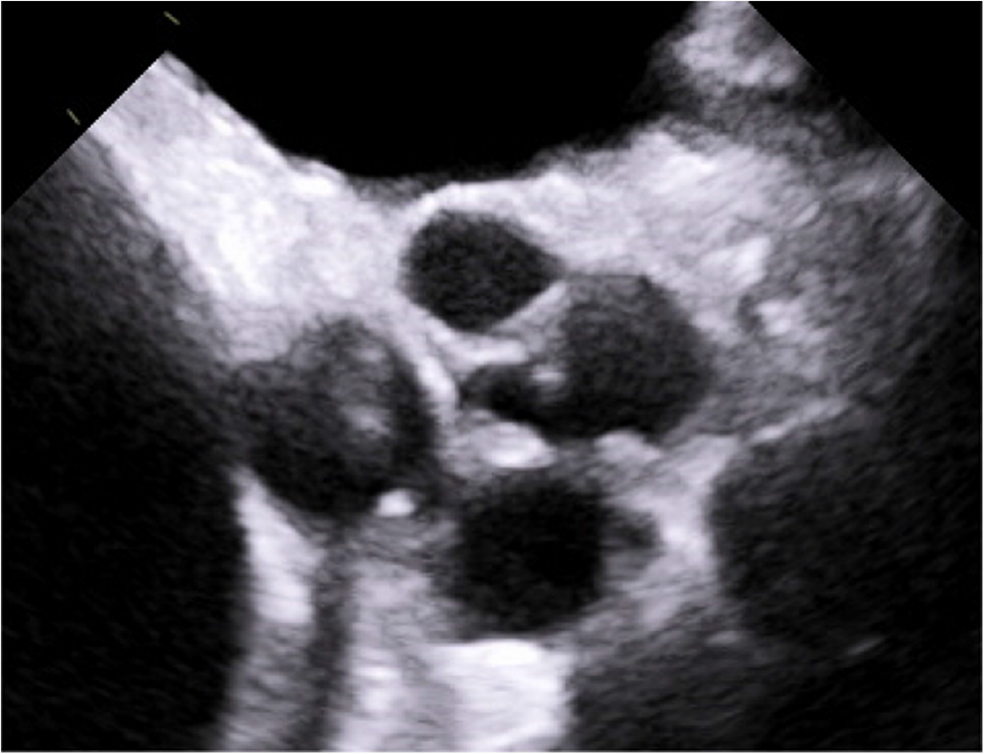
TEE short axis view. The typical configuration of the quadricuspid aortic valve with four commissures is readily visible

Unicuspid aortic valves are best diagnosed through their eccentric opening seen in systole (Fig. 4). Experience shows that the vast majority of unicuspid aortic valves are misdiagnosed as bicuspid, both by echocardiography [56] and in the operating room [48]. Failure to diagnose this morphology correctly may lead to wrong decision-making. Finally, the quadricuspid aortic valve must be detected (Fig. 5).

**Fig. 6:**
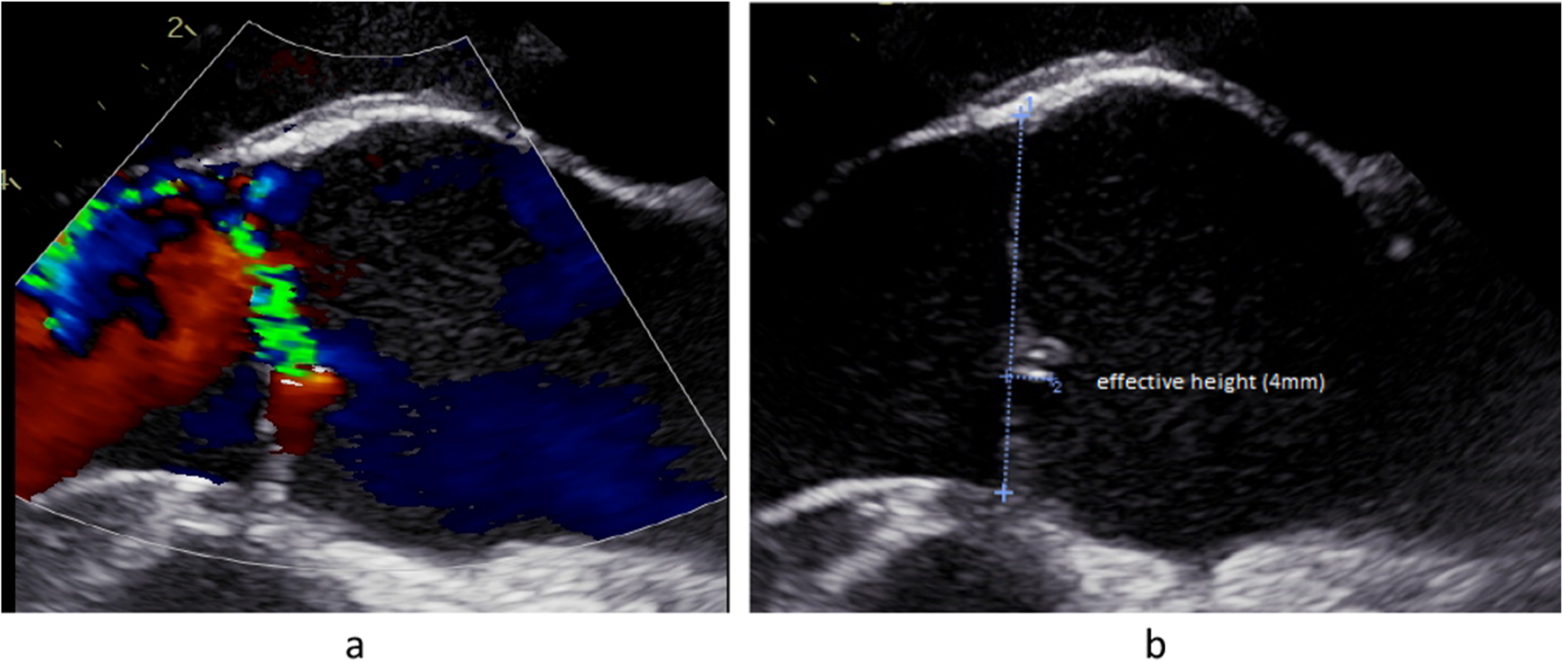
TEE long axis view. **a** The eccentric jet towards the anterior septal wall is suggestive of prolapse of the right cusp. **b** Prolapse is visible and quantified by measuring an effective height of the right cusp of 4 mm

Imaging should also define the *cusp pathologies* that require repair and ideally those that are better treated by replacement. Prolapse can be assumed by eccentricity of the regurgitant jet (Fig. 6a). It can be documented by measuring a low effective height (< 8 mm) in the absence of cusp retraction (Fig. 6b). Two-dimensional echocardiography is relatively reliable for the analysis of the right cusp but less so for the noncoronary cusp; the left cusp can rarely be visualized by two-dimensional imaging. For precise analysis of all three cusps, three-dimensional echocardiography is indispensable [37••]. Contrary to other opinions [57], we have not been able to visualize fenestrations directly. Fenestrations may be suspected in some instances of partial prolapse and are a differential diagnosis of floating tissue suggestive of smaller vegetations. Perforations are sometimes visualized directly as a tissue defect; more often, a jet is visible arising from the body of a cusp.

**Fig. 7:**
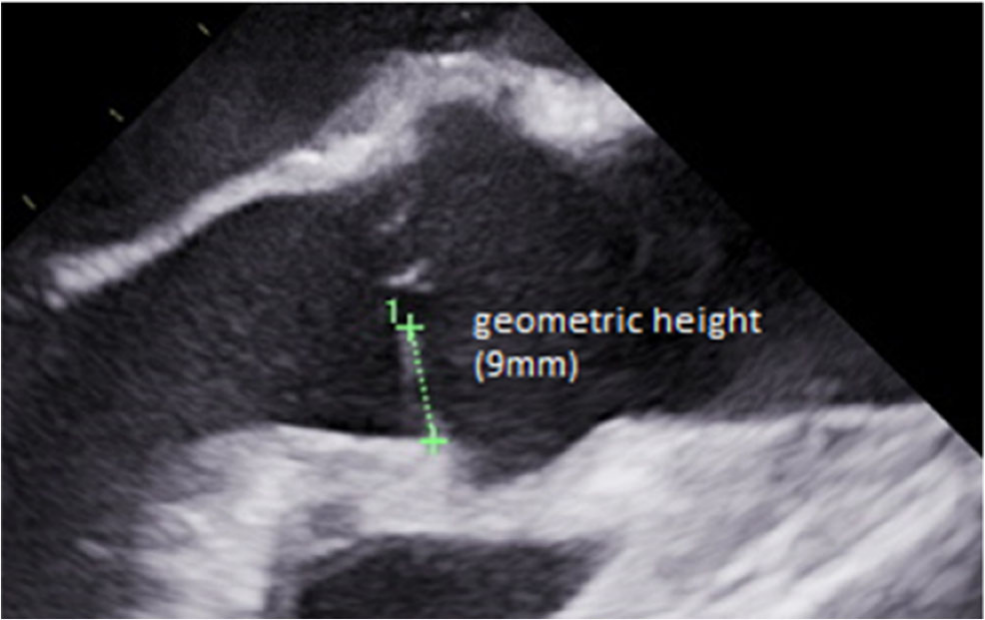
TEE long axis view of a tricuspid aortic valve. Visual assessment suggests cusp retraction. The determination of geometric cusp height shows a height of 9mm, which is far too low for a normal cusp. Thus, the mechanism of regurgitation can objectively be classified as retraction

The typical echocardiographic pathology of active endocarditis is well known [58]. Calcifications are easily visible on echocardiography if the whole valve is carefully examined. Retraction may be assumed by the visual impression of cusps that are too short for adequate adaptation in a long axis view (Fig. 7). A more objective and quantitative approach is the measurement of geometric height [36, 59] of the cusp (Fig. 7). Echocardiographic measurements are often 2 to 3 mm shorter than intraoperative determination. As with prolapse, three-dimensional imaging is necessary for the precise analysis of all three cusps [37••].

## Conclusion

In summary, many regurgitant aortic valves can be repaired using current techniques. Repair is possible for all variants of aortic valve morphology, most frequently in tricuspid and bicuspid aortic valves. The decision for repair has to be made more carefully in a unicuspid anatomy. Likewise, the aortic valve can be preserved or repaired in most cases of root aneurysm independent of the preoperative degree of regurgitation.

Cusp pathology is always present in isolated regurgitation and is frequent in root aneurysm. Of the different pathologies, prolapse can be corrected reproducibly and with good long-term results if the repair strategy is guided by intraoperative measurement. Cusp calcification, retraction, or perforation in the presence of active endocarditis are poor substrates for repair and should lead to replacement as the primary option. Modern imaging can principally identify such pathologies, at least if three-dimensional imaging including TEE is employed.
